# A global neuronopathic gaucher disease registry (GARDIAN): a patient-led initiative

**DOI:** 10.1186/s13023-023-02828-w

**Published:** 2023-07-21

**Authors:** Tanya Collin-Histed, Madeline Stoodley, Kathleen Beusterien, Deborah Elstein, Dena H. Jaffe, Shoshana Revel-Vilk, Elin Haf Davies, the International Gaucher Alliance (IGA)

**Affiliations:** 1 International Gaucher Alliance (IGA), 86-90 Paul Street, London, EC2A 4NE UK; 2 Cerner Enviza, an Oracle Company, 51 Valley Stream Pkwy, Malvern, PA 19355 USA; 3 Jerusalem, Israel; 4 Gaucher Unit, Shaare Zedek Medical Center, Jerusalem, Israel; 5 Faculty of Medicine, Hebrew University, Jerusalem, Israel; 6 Aparito, 11-12 Gwenfro Technology Park, Croesnewydd Road, Wrexham, UK; 7 International Gaucher Alliance, 86-90 Paul Street, London, EC2A 4NE UK

**Keywords:** Registry, Rare disease, Neuronopathic gaucher disease, GARDIAN, Patient-reported outcomes, Observer-reported outcomes, PRO, ObsRO

## Abstract

**Background:**

Gaucher disease (GD) is a rare autosomal recessive lysosomal storage disorder. GD types 2 and 3 are known as neuronopathic Gaucher disease (nGD) because they have brain involvement that progresses over time. Implementing a systematic approach to the collection of real-world clinical and patient-relevant outcomes data in nGD presents an opportunity to fill critical knowledge gaps and ultimately help healthcare providers in the management of this patient population. This paper summarizes the development of a patient-initiated Gaucher Registry for Development Innovation and Analysis of Neuronopathic Disease (GARDIAN).

**Methods:**

The International Gaucher Alliance led the GARDIAN planning, including governance, scope, stakeholder involvement, platform, and reporting. Registry element input was determined in a series of meetings with clinical experts, patients, and caregivers, who identified key clinical variables and the draft content of nGD patient-reported outcomes (PRO) and observer-reported outcomes (ObsRO) focusing on symptoms, patient physical and emotional functioning. These were then tested in cognitive interviews with patients with nGD (> 12 years of age) and caregivers.

**Results:**

Core registry data elements (n = 138) were identified by seven global clinical experts from Egypt, Germany, Israel, Japan, United Kingdom (UK), and United State (US) and reviewed via online Delphi method by 14 additional clinicians with experience of nGD from six countries and three pharmaceutical representatives. The elements were consistent with those identified via interviews with 10 patients/caregivers with nGD from Japan, Sweden, UK, and US. Key domains identified were demographics, diagnostic information, health status, clinical symptomatology, laboratory testing, treatment, healthcare resource utilization, aids/home improvements, and patient/caregiver burden and quality of life, specifically physical functioning, self-care, daily and social activities, emotional impacts, support services, and caregiver-specific impacts. Nine caregivers and six patients from the US, UK, China, Mexico, Egypt, and Japan participated in the cognitive interviews that informed revisions to ensure that all items are understandable and interpreted as intended.

**Conclusions:**

The comprehensive set of clinical and patient relevant outcomes data, developed collaboratively among all stakeholders, to be reported using GARDIAN will bridge the many gaps in the understanding of nGD and align with regulatory frameworks on real-world data needs.

## Background

Neuronopathic Gaucher disease (nGD) is a rare disease occurring in between 0.17 and 0.55 per 100,000 live births [1]. The nGD type 2 (GD2) and type 3 (GD3) forms of this autosomal recessive lysosomal storage disorder are distinguished from the substantially more prevalent GD type 1 (GD1) with early onset central nervous system involvement [2]. Currently, therapies approved for use to treat GD2 and GD3 rely primarily on enzyme replacement therapy to treat skeletal and systemic GD manifestations. Potentially curative neurologic treatments that cross the blood brain barrier, including brain-penetrating substrate-reduction therapy (SRT), chaperone therapy, and gene therapy, are currently in development [3–5].

A key treatment barrier for people with nGD and other rare diseases involves the drug development and approval process [6–8]. However, progress has been made globally regarding the regulatory framework for drug approval for orphan drugs. For example, in 2018, the United States Food and Drug Administration (FDA) published its *Framework for FDA’s Real-World Evidence Program* [9] to guide researchers and regulators toward the use of real-world data. As noted by the FDA, real-world data could be used for improving efficiency of clinical trials in rare diseases by aiding in patient recruitment through identifying geographically distributed research cohorts, and real-world evidence (RWE) can be leveraged to support drug product approvals with synthetic control arms. In Europe, policies related to treatment and care of patients with rare diseases have been developed, including the creation of the European Reference Networks (cross-border collaboration for diagnosis, treatment, and care of patients with rare diseases), Orphanet (repository of information about rare diseases), and the European Organisation for Rare Diseases (EURORDIS; patient advocacy for rare disease at the European level) [8].

Regulators, health technology assessment bodies, and pharmaceutical companies realize the necessity for patient and public involvement (PPI) or patient group engagement in clinical research, in general [10–12] and for rare diseases, specifically [13, 14]. PPI is becoming acceptable practice with recognized value, for example, empowering patients and efficiencies in product development and commercialization [12, 15]. Real-world data from the patient perspective, including patient-organization registries, present an opportunity to fill critical knowledge gaps, such as providing data on the epidemiological and natural history of disease and informing researchers and healthcare providers of important concerns and outcomes [9, 11, 12, 16–18].

To this end, the International Gaucher Alliance (IGA), which represents the interests of patients with GD and of non-profit GD patient groups, initiated the development of a global nGD patient registry. The design of the registry, propelled by patients and their caregivers and in collaboration with physicians, academics, and industry representatives, aims to collect disease, treatment, management, and outcome data to inform research, therapeutic development, and clinical care.

This paper summarizes the planning and the elements of the Gaucher Registry for Development Innovation and Analysis of Neuronopathic Disease (GARDIAN), a global nGD patient-driven disease registry.

## Methods

### Registry planning

**Fig. 1:**
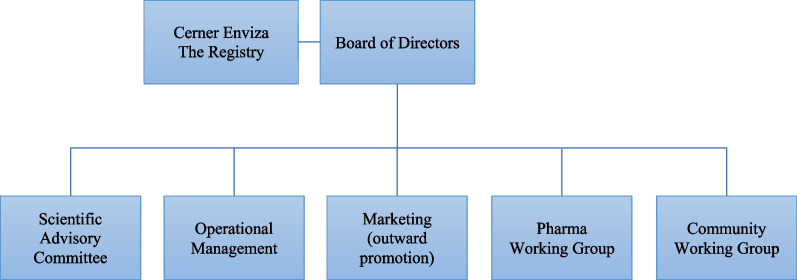
GARDIAN governance schema

The planning and governance of the GARDIAN were led by the IGA following the best practices described by EURORDIS and the US Agency for Healthcare Research and Quality (AHRQ) [18, 22]. During a series of IGA meetings, the goals, governance, scope, data sources, stakeholder involvement, platform, and reporting were determined (Fig. 1). The governance board of directors is responsible for GARDIAN oversight, holds all legal responsibility, and owns all data emerging from the registry. Cerner Enviza, an Oracle company, was involved in the development, planning, and implementation of the goals alongside the governance board and is responsible for the day-to-day operations. Aparito was responsible for creating the GARDIAN platform. Two committees were established, operational management and marketing, to address the day to day operational and legal responsibilities and outward registry promotion. Members of these committees are IGA employees and the directors of the IGL board. The IGL directors are all volunteers and include a caregiver, two patient advocates, a financial businessman, a clinician and a professional with experience of working in the rare disease pharmaceutical environment. Three working groups were created to address the three primary facets of GARDIAN—scientific advisory, pharmaceutical industry, and community. These working groups include representatives from the IGA, patients, caregivers, ethicists, clinical experts, researchers, representatives of the pharmaceutical industry and registry managers. The role of the scientific advisory committee is to advise the governance board on scientific and clinical matters, consider and approve research and data requests, and contribute to the ongoing development of GARDIAN for the benefit of the global patient community. The role of the pharmaceutical industry working group is to regulate the relationship with pharmaceutical and biotech industries, define rules for sponsorship opportunities, define access and scope of access to non-identifiable, aggregated data stored and managed by GARDIAN, and approve and/or partner with these organizations for scientific publication aligned with the main objectives of GARDIAN. The role of the community working group is to be the patient and caregiver voice on GARDIAN, making research recommendations to the scientific advisory board, provide feedback on GARDIAN’s accessibility, website content, and the dissemination of information to the patient community.

All members of the governance board and working group members are required to sign a code of conduct, have terms of reference, and complete a statement of duality of interest, if applicable. The term of each member is three years.

Clinical and patient review processes were created to establish content validity, appropriateness, and comprehensiveness of the core data elements to be collected in the registry. Per the United Kingdom (UK) National Institute for Health Research (NIHR), Ethics approval was not required for this activity, which was to ensure that design of the GARDIAN involves expert, patient, and caregiver involvement [23].

The data and collection process of this registry will be compliant with data collection and sharing regulations, including General Data Protection Regulation (GDPR) [19], Health Insurance Portability and Accountability Act (HIPAA) [20], and US FDA Part 11 [21], with intent to solicit reviews from European Medicines Agency (EMA) and the FDA.

## Registry input

### Identification of core data elements

Clinical experts as well as caregivers and patients were consulted regarding the clinically relevant and usable data capture fields that should be collected as part of the registry. First, the IGA drafted a template of potential clinical data capture fields. Second, the IGA identified and invited an international group of clinicians and clinical researchers in the field of nGD to participate in a meeting (face-to-face or remote) to further refine the initial set of variables. The participants included seven clinical experts from Egypt (n = 1), Germany (n = 1), Israel (n = 1), Japan (n = 1), UK (n = 2), and the United State (US) (n = 1). The IGA sponsored participants’ expenses for those who were able to attend in person. The meeting took place on March 20, 2019, at the Royal Free Hospital, London, UK.

These variables were then populated into an online document for verification by a broader clinical expert community with experience of nGD (14 treating physicians from six countries (France, Japan, Pakistan, Spain, UK, and US) and three pharmaceutical representatives). The verification involved an online Delphi method to reach consensus for the proposed common data set. Data fields that were scored as disagree/strongly disagree by three or more clinical experts were deleted from the list.

Experienced qualitative researchers (MS, EHD) conducted interviews with caregivers and patients to confirm key disease outcomes that should be considered as data elements for the registry. A total of ten individuals provided feedback: three patients with GD3 and seven caregivers (two fathers, five mothers) of children with GD2 (n = 1) and GD3 (n = 3). They lived in Japan, Sweden, UK, and US of which seven were Caucasian and four were Asian. The age range of the individuals living with nGD ranged from 2.5 to 25 years of age. The caregivers and patients were recruited by the IGA via online social media announcements (Facebook and Twitter) to ensure a global reach, although recruitment was limited to English-literate individuals, and were offered £80 Amazon vouchers for taking part in the interviews. Confirmation of confidentiality was discussed, and verbal consent was obtained at the beginning of each interview. The interviews were held online using Zoom video link, unless the interviewer requested that the interview be conducted in person, and their request could be accommodated logistically.

Clinical outcome assessments (COAs) were developed to describe how a patient with nGD feels and functions. A co-author (DE) developed the initial format and questions and donated them to the IGA for use in developing the PRO and ObsRO for the registry. Using FDA-based guidance [24, 25], two types of COAs were developed: a neuronopathic-specific patient reported outcomes measure (nGD-PRO) and observer reported outcomes measure (nGD-ObsRO). A PRO is any report of the status of a patient’s health condition that comes directly from the patient, without interpretation of the patient’s response by a clinician or anyone else. An ObsRO is a measurement based on a report of observable signs, events or behaviors related to a patient’s health condition by someone other than the patient or a health professional.

### Cognitive pre-test interviews of nGD-PRO and nGD-ObsRO

Cognitive interviews were performed with caregivers and patients to obtain feedback on the draft nGD-ObsRO and nGD-PRO, respectively. The interviews focused on the clarity, relevance, and comprehensiveness of the content, including appropriateness of language. The interviews were conducted by trained moderators via telephone. The moderators used a screen-sharing platform to share the questionnaires with the participants, who viewed them on their devices. A think-aloud approach was used where the participant read each question and provided their thoughts regarding the meaning of the question and their interpretation of how they would answer it. All interviews were transcribed, and the transcripts were uploaded into NVivo qualitative analysis software for analysis.

Two waves of interviews were conducted: first, the English versions of the nGD-ObsRO and PRO were conducted in the UK and US. Based on the findings, the English draft versions were revised, and translations were developed for all participating countries. For each language version, two native speakers translated each questionnaire and convened a reconciliation meeting to refine and finalize the draft translated versions. The final draft translated versions were then used in the cognitive debriefing interviews in each respective country. All participants provided informed consent and were compensated for their time as per country appropriate rates. The cognitive interview protocol was reviewed and received Exemption status from Pearl IRB, 05 March 2021.

## Results

### Core data elements

Based on the initial clinical expert meeting, 138 possible data capture fields were identified and included for the subsequent Delphi review. Table 1 lists the key demographic and clinical data domains proposed by the key opinion leaders (KOLs) for the GARDIAN. There was consensus on the removal of ten initially proposed variables (country of birth, country of residence, nerve conduction velocity/ electromyography indication for test), electrocardiogram, cerebrospinal fluid testing, gastrology and general surgery, dyspepsia, constipation, and reflux. Although not all agreed, the proposed data variable, transfer capacity of the lung TLCO gas transfer exchange, also was removed.

**Table 1:** Key demographic and clinical data domains for the neuronopathic Gaucher disease registry

Domains	Example items
Demographics	Age, sex, ethnicity, self-reported heritage/ancestry (maternal and paternal), consanguinity, educational achievements and abilities, working status, living arrangements, family history of Parkinson’s disease
Diagnostic information	Gaucher type, age at diagnosis (any type), genetic testing performed for diagnosis, genetic and phenotypic profile, symptoms and severity at diagnosis, other testing at diagnosis (e.g., neurological, laboratory), saccades and mSST
Health status	Body mass index, fertility status, physical and mental health status
Clinical symptomatology	Symptoms (e.g., developmental, hematological, neurological, respiratory, skeletal)
Testing	Laboratory tests (e.g., BFC, Hb, platelets, chitotriosidase, and imaging (e.g., DEXA, spleen ultrasound, EEG, brain MRI, spinal x-ray, chest CT)
Treatment	Prescription medication (type, dose, adverse events), supplements, procedures/surgery (tracheostomy, splenectomy)
Healthcare resource utilization	Specialist visits and hospitalizations
Aids and home improvements	Aids (e.g., glasses, hearing aids, canes) and home improvements (e.g., ramps, stair lift, walk-in shower)
Patient/caregiver burden and quality of life	Symptoms (e.g., pain, fatigue, anxiety, depression, sleep), physical function, self-care, social and emotional impact

BFC, blood full count; CT, computed tomography; DEXA, dual-energy x-ray absorptiometry; EEG, electroencephalogram; Hb, hemoglobin; MRI, magnetic resonance imaging; mSST, modified Severity Scoring Tool [26]

Additional recommendations from KOLs were that the GARDIAN should use a common coding standard or ontology to create a lean and simple data set that is compliance eligible. Also, that a new neuronopathic-specific severity scoring scale should be developed that incorporates and measures both the visceral and neurological manifestations of nGD.

The symptoms and impacts identified fall into seven domains: nGD symptoms, physical functioning, self-regulation, socio-emotional functioning, support services, and caregiver-specific impacts as described below (Table 2). The feedback received were that having bone deformity and tremors, including seizures or fits, are core symptoms that can affect movement and have wide-ranging impacts. Studies have emphasized the frequent and disabling nature of bone-related pathology, including frequent bone crises, impaired gait, and parkinsonian symptoms [27–29]. Pain and discomfort can be a consistent part of daily life. Symptoms can vary widely across patients, where some individuals are very limited in their mobility, whereas others are able to live independently with some support from personal assistants and nurses. Cognitive impacts revolve around inability to focus and feeling overwhelmed with multiple things to process at one time. Lack of balance, tremors, and fatigue, have impacts on physical functioning, including walking, and sitting. Fine motor skills sometimes prove to be problematic; some individuals require assistance when dressing, bathing, using utensils, or preparing food. Many patients struggle to make friends, and they may only interact with family members and staff in the medical community. This can lead to feeling isolated and socially excluded. Resulting emotional impacts may include anxiety, depression, and stress. Nevertheless, patients may also show resilience whilst facing physical and psychological difficulties.

**Table 2:** Neuronopathic GD symptoms and impacts identified by clinical experts and caregivers/patients

Concept	Clinical expert input	Patient/caregiver input
*Symptoms*		
Fatigue	X	X
Pain in small joints	X	
Pain in large joints	X	
Salivation/drooling	X	X
Chewing/swallowing/choking	X	X
Bone/muscle pain, including back pain	X	X
Nerve pain	X	X
Balance/coordination/feel wobbly	X	X
Seizure/jerky movements	X	X
Impaired speech		X
Trouble concentrating/staying focused		X
Feeling overwhelmed/memory trouble		X
*Physical Function*		
Sitting	X	X
Using utensils/Writing	X	X
Walking	X	X
Reaching for objects independently	X	
Performing hobbies		X
*Self-Regulation*		
Dressing	X	X
Hygiene	X	X
Toileting	X	
Sleeping	X	X
Prepare food		X
*Socio-Emotional Functioning*		
Attending school/work	X	X
Recognizing significant others	X	X
Feelings isolated/lonely	X	X
Irritability/anger	X	X
Anxiety	X	X
Happy/unhappy	X	X
School/work stress		X
Stressful traveling outside of home		X
Concern about treatment side effects		X
Concern about caregiver burden	X	
*Support Services/Coping*		
Receiving physical support services	X	X
Accommodations made at school/work		X
Receiving/using emotional support		X
Connected to others with Gaucher Disease		X
Supportive healthcare providers		X
Clinical study participation		X
*Caregiver Impacts*		
Stress in caregiving	X	X
Fatigue	X	X
Anxious about patient’s health	X	X
Positive/hopeful about patient’s health	X	X
Stress in traveling with patient		X

These outcomes informed the content of a draft nGD-PRO and nGD-ObsRO. It was determined the nGD-PRO can be administered to patients who are at least 12 years of age and whose caregiver affirmatively responds to the question: Is the child you care for with Gaucher disease able to answer questions about how they feel and function? The nGD-ObsRO items focus on the same patient outcomes captured in the nGD-PRO; however, because the nGD-ObsRO captures only patient outcomes that can be observed, each item within this measure asks the caregiver to provide a response “based on what you observed or what the patient told you.” The response scale for all of the items is the 11-point numerical rating scale, where respondents can choose a number between 0 and 10. To enhance interpretation of the numbers, descriptive categories are included along the scale.

### nGD PRO and nGD ObsRO cognitive interview findings

A total of nine caregivers and six patients from the US, UK, China, Mexico, Egypt, and Japan participated in the cognitive interviews (Table 3).

**Table 3:** Participants of the nGD PRO and nGD ObsRO cognitive interviews

Subject (Residence [# participants])	Patient age	Patient gender	Caregiver relation (age)
United Kingdom (3 CGs, 2 PTs)			
CG_UK_01	11 years	Female	Mother (36 years)
CG_UK_02	24 years	Male	Father (63 years)
CG_UK_03	4 years	Male	Mother (36 years)
PT_UK_01	24 years	n/r	n/a
PT_UK_02	26 years	n/r	n/a
China (2 CGs, 1 PT)			
PT_CH_01	25 years	Male	Father (53 years)
CG_CH_01	13 years	n/r	Father (38 years)
CG_CH_02	11 years	Male	Mother (42 years)
Egypt (1 CG, 1PT)			
CG_EG_01	14 years^a^	Male	n/a
PT_EG_01	14 years^a^	Male	Mother (42 years)
Mexico (1 CG, 1 PT)			
PT_MX_02	16 years^b^	Female	n/a
CG_MX_01	16 years^b^	Female	Father (59 years)
Japan (1 CG)			
CG_JP_01	7 years	Male	Father (40 years)
United States (1 CG)			
CG_US_01	13 years	Male	Mother (50 years)

^a^Same patient

^b^Same patient

CG, Caregiver, ObsRO, Observer reported outcome, n/a, Not available, nGD, Neuronopathic Gaucher disease, n/r, Not reported, PRO, Patient reported outcome, PT, Patient

Overall, the PRO and ObsRO were well-received. As noted by the moderator in China: “*The caregivers are very grateful that we can pay attention to this disease, and they think that the questionnaire is very comprehensive and asks about the things that patients usually experience.”* In the first wave of cognitive interviews (Wave 1), the English versions of the nGD-PRO and nGD-ObsRO were tested in the US and UK. Based on the feedback in Wave 1, translations were developed of the PRO and ObsRO for each target language: Spanish, Arabic, Japanese, Simplified Chinese, French, and German. In Wave 2, the Spanish, Arabic, Simplified Chinese, and Japanese versions were tested in Mexico, Egypt, China, and Japan, respectively. Revisions to the measures based on the findings from Waves 1 and 2 are summarized in Table 4. Although no participants were recruited from France or Germany, the French and German translated versions were revised accordingly based on the feedback from the other participating countries.

**Table 4:** Key item revisions made based on cognitive interviews

Original	Issue	Revision
*First Wave (English versions tested in US and UK)*		
Recall period for all items is “last 7 days”	A longer recall period would more accurately reflect patient experience	Recall period for all items revised to “last 14 days”
On average, how much nerve pain (e.g., feeling pins and needles, sharp tingling or numbness) did the patient have over the last 7 days based on what you observed or what the patient told you?	The term “nerve pain” did not resonate with participants; they identified with “feeling pins and needles and numbness”	Did the patient have numbness or pins and needles the last 14 days based on what you observed or what the patient told you?
A few caregivers noted that the patients had to stop and rest after walking for a select period	New concept raised: needing to rest after walking	Did the patient need to rest after standing or walking for a period of time over the last 14 days
How much drooling/salivating or secretions have you experienced over the last 14 days?	PTs and CGs and PTs indicated that ‘dribbling’ was more aligned with disease impact versus ‘salivating’	Did you have drooling or dribbling over the last 14 days?
Did you feel overwhelmed by too much information and/or had trouble remembering what comes next over the last 14 days?	Recommend asking only about trouble with memory as feeling overwhelmed can be interpreted as separate and being related to feeling stressed	Did you have trouble remembering things over the last 14 days?
*Second Wave (Simplistic Chinese, Spanish, Arabic, and Japanese versions)*		
Have you needed assistance sitting upright as in a chair over the last 14 days?	Suggested removing ‘upright’ as some patients cannot physically sit upright because their back is deformed due to the disease	Have you needed assistance sitting in a chair over the last 14 days?
How often did you prepare food or cook food without help over the last 14 days?	Suggestion to be more specific about needing help as well as type of meal	Overall, did you need help preparing simple meals (for example, making a sandwich) over the last 14 days?
Did you have difficulty performing any hobbies or your daily activities, including using a computer/iPad over the last 14 days?	This item is asking about multiple concepts, i.e., sports and daily activities; in addition, using a computer not considered hobby	Did you have difficulty performing your choice of hobbies or activities over the last 14 days?
Did you feel happy or positive over the last 14 days?	The word ‘positive’ was not well understood among participants across countries	Did you feel happy over the last 14 days?

Based on Wave 1, the original reference period of the last 7 days was extended to the last 14 days to capture outcomes more comprehensively. One caregiver indicated that the last 7 days is not a sufficient time period to characterize disease impacts, one noted that the patient’s infusions are a few weeks apart and thus a longer reference period would more accurately reflect treatment impacts, and one felt a longer time period was optimal because some of the questions ask about what the patient was feeling.

Wave 1 also informed item-specific revisions. For example, an item asking about nerve pain was revised to refer only to ‘pins and needles and numbness’; the term ‘nerve pain’ was removed because this was not well understood: *“I think the tingling and numbness is easier to understand, but nerve pain is a little difficult” (CG_UK_01); “When he has seizures, he definitely has this type of pain [pins and needles]” (CG-UK02)*. In addition, a few caregivers noted that the patients may not need help walking, but they may need to rest after walking for a period of time: *“It’s walking for long distance, he doesn’t need help, but he needs rest” (CG_UK_01)*. Thus, a new item was added to capture the need to rest with standing or walking. The symptoms captured in the measures include fatigue; bone, joint, or back pain; and numbness or pins and needles. Although all of the ObsRO items have an option for ‘did not observe/did not appear to have’ that can be endorsed as appropriate, the caregiver participants generally found it easy to report on the magnitude of these symptoms experienced by the patient*: “Nine points, he is very tired” (CG_CH_01); “He really doesn’t feel fatigued unless he does physical activity” (CG_MX_01); “He was fine when he got up in the morning. In the afternoon, the teacher told me that he felt very tired when he was in class and when he came back in the evening” (CG_CH_02); “He wakes at night with pains in his legs” (CG_UK_03). “He tells me that his leg hurts when he feels pain” (CG_EG_01); “the knee [is where he has numbness or tingling]” (CG_CH_01); “He has a very painful back, 9 points. Because my son has osteoporosis, his waist hurts every day when he gets up. He just talks about pain every day” (CG_CH_01);“Three points; Because he used to have severe pain, now he has taken [medication], and now he has relieved a lot. I used to massage his legs every night, and now his pain has eased a lot” (CG_CH_02).*

The two cognitive symptom items, ‘trouble concentrating or staying focused’ and ‘trouble remembering things’, were relatively easy to answer for the caregivers: *“He stays focused for only short periods of time” (CG_UK_01); “He is very good at concentration and memorizing” (CG_EG_01); “He has a little difficulty, especially when doing homework. The teacher also reported that he was unable to concentrate during class…He plays with the pen and bites the pen” (CG_CH_02); “He forgets very quickly in his studies. I dictate Chinese with him every night. When he dictates the next night, he forgets it again. I dictated to him repeatedly, but he couldn't remember it” (CG_CH_02); “I would choose 2; at times, she is very focused on what she was initially doing, but she is very easily distracted.” (CG_MX_01).*

The caregivers also found it easy to report on the patient’s emotions; the measures capture being anxious or panicky, frustrated or angry, happy, and sad. The caregivers were highly sensitive to the patients’ feelings: *“My daughter is very cheerful, and so, when she’s anxious or she feels worried, or she’s scared, or feels panic, you start noticing her being nervous, she looks to one side and the other, she tells you what she feels herself. Or we see that she’s anxious, that she’s nervous; she starts pinching her fingers, pulling her hair, that is, those are reflexes she has as a reaction to fear…It’s very easy to spot her moods.” (CG_MX_01); “I would say most of the time he’s happy” CG_UK_01; “But then he has his moments where he’s having a tantrum and he’s miserable” (CG-UK03); “When we walk out of our house, he puts his hands over his ears because he doesn’t like to hear the sound of cars going past” (CG-UK03).*

The final measures comprise 33 items that focus on the patient’s symptoms, functional status, and well-being. Additionally, the ObsRO includes items focusing on caregiver impacts. Many of the cognitive interview participants confirmed the relevance of the items: *“The symptoms of this disease are basically in it. The questions asked by the doctor are the same as your questionnaire.” (CG_CH_01); “This questionnaire is quite comprehensive.” (CG_CH_02); “It’s very interesting because these are questions that we are not normally asked; others always focus on the beginning, and not on what they have been living through in the last 14 days.” (CG_MX_01).*

## Discussion

The development of the IGA’s patient-initiated nGD registry, GARDIAN, focused on the patient and caregiver’s experience and burden and utilized multi-stakeholder involvement to strengthen and ensure its effective use in patient management and treatment development. These first steps highlighted the unmet needs in the understanding and management of patients with nGD and in providing the necessary physical, psychological, and social supports to reduce the disease burden and empower the patient and caregiver [30–33].

Among the numerous advances in GD research is the identification of over 350 ẞ-glucocerebrosidase mutations and the approval of GD1 treatments. Still, modest achievements for those with nGD have occurred over this same period and only recently was a consensus definition for GD2 and GD3 published [2]. This consensus definition satisfies a critical element for improving the standard of diagnosis, care, and research. This consensus definition, together with GARDIAN, can provide evidence to define the natural history and severity of the disease, as well as validate outcomes that appropriately measure patient and caregiver burden. Further, the broad-based support and consensus for GARDIAN, provided by clinicians and the pharmaceutical industry, is a clear indication of the existing knowledge gaps that require a centralized effort to collect data for this rare disease [27–29].

Engagement of KOLs in the process of developing the GARDIAN was important for establishing a list of key demographic and clinical data domains. Having bone deformity and tremors, including seizures or fits, are core symptoms that can affect movement and have wide-ranging impacts. Studies have emphasized the frequent and disabling nature of bone-related pathology, including frequent bone crises, impaired gait, and parkinsonian symptoms [27–29]. Interestingly, clinicians and patients differed somewhat with regard to defining symptoms and impact of disease. For example, clinicians noted joint pain as symptoms of importance whereas patients noted speech, concentration, and memory issues. More pronounced, however is the impact on support services/coping that were highlighted by patients and less so by clinicians. These finding reinforce the importance of the patient voice in healthcare.

There remains a paucity of literature examining the psychosocial impact of nGD on the patient or those caring for them. Qualitative studies have noted the challenge of coping with this diagnosis, its impact on personal and professional roles, and the associated anxiety and mood symptoms [35]. Importantly, patients also report that there have been positive aspects to their illness, including strengthening family relationships. Further studies have identified the potential impact of somatic concerns and depressed mood [36] among patients, as well as the significant impact that caregiving for those with this disease can have on quality of life [37, 38]. In our study, however, the number and geographic diversity of patients and caregivers interviewed were limited. For this reason, a patient registry, such as GARDIAN, can provide further validation of the complexity of nGD.

Nonetheless, based on the cognitive interview pre-testing of the nGD PRO and nGD ObsRO, the content of these measures was shown to be robust and comprehensive in capturing the key outcomes of nGD. It is apparent that the nGD community would benefit from having additional peer-to-peer and specialist support, especially in having ongoing discussions about palliative care. End-of-life discussions with the patient and their family need to become a routine part of care planning and available support services. However, without adequate clinical knowledge, patients and caregivers may not be able to clearly communicate their needs or could have unrealistic expectations regarding prognosis.

Future efforts are therefore needed to provide novel forms of support and public engagement in the context of nGD. The rare nature of this condition and associated caregiver burden and patients’ impaired mobility could make utilising on-line supportive interventions, with moderated and supported conversations and topics of interest, a potentially valuable source of peer support and friendship for some people. Further, IGA initiatives are underway to enable patients diagnosed with nGD and their caregivers to engage with the public and to share their experiences of living with this disease. Such public engagement can not only increase awareness of this disabling disease among healthcare professionals and the public at large, but also facilitate peer support efforts.

In addition, initiatives have been developed to promote patient and caregiver stakeholders to become partners and leaders regarding their own health or the health of their loved one [39–43]. The INVOLVE initiative, for example, lays the foundation for PPI in clinical research, guideline development, and patient engagement based on respect, support, transparency, responsiveness, fairness of opportunity, and accountability [42], while the Guidance for Reporting Involvement of Patients and the Public version 2 (GRIPP2) checklist was developed to provide internationally acceptable, standardized guidelines for PPI reporting [41].

The goals and design of GARDIAN will provide novel insight into GD and help enhance our current understanding of the disease, its treatment and care, and guide future nGD priorities in research and supportive care development. Notably, data from GARDIAN can help support the further development of a psychometric assessment of the draft nGD PRO and nGD ObsRO. The use of such scales, to be validated for use in the GARDIAN, will enable the measurement of patient relevant endpoints, help ensure a comprehensive measurement strategy for assessing health-related quality of life outcomes, and provide an evidence-based approach to addressing the burden of nGD across all age groups. Existing GD registries that focus primarily on GD1, such as the International Collaborative Gaucher Group (ICGG) [44] and Gaucher Outcome Survey (GOS) [45] could be used to complement the data from GARDIAN to understand differences between disease types.

## Conclusion

The IGA is committed to addressing the significant unmet needs in nGD and has identified the value that the GARDIAN registry and the development and validation of a nGD-specific PRO and ObsRO can offer in this regard. The IGA is best placed to support this effort with an emphasis on secure data management and patient-centered data collection. These patient and caregiver reported data will bridge the many gaps in the understanding of neuronopathic GD and align with regulatory frameworks on real world data.

## Data Availability

The data analyzed during the current study and supporting Tables 2–4 are not publicly available in order to protect patient privacy. Data requests can be made to the International Gaucher Alliance (IGA) admin@gaucheralliance.org.
